# Competition Elicits more Physical Affiliation between Male than Female Friends

**DOI:** 10.1038/s41598-018-26544-9

**Published:** 2018-05-30

**Authors:** Joyce F. Benenson, Maxwell M. White, Delfina Martinez Pandiani, Lindsay J. Hillyer, Sera Kantor, Henry Markovits, Richard W. Wrangham

**Affiliations:** 1 Department of Psychology, Emmanuel College, Boston, 02115 USA; 2 Department of Human Evolutionary Biology, Harvard University, Cambridge, 02138 USA; 3 Département de Psychologie, Université du Québec à Montréal, Montréal, H3C 3P8 Canada

## Abstract

Across species, cooperative alliances must withstand internal tensions. The mechanisms by which allies respond to competing against one another have been studied extensively in non-human animals, but much less so in humans. In non-human species, affiliative physical contact and close proximity immediately following a contest are utilized to define reconciliation between opponents. The proportion of conflicts that are reconciled however differs markedly by species and sex. The purpose of this study was to examine whether, like many other social species, humans utilize physical contact and close proximity following a competition between friends, and if so, whether one sex is more likely to exhibit these behaviors. Using a standardized procedure, two same-gender friends competed against one another producing a clear winner and loser. Prior to and following the competition, the friends relaxed together. Videotapes of the relaxation periods showed that male friends spent more time than female friends engaged in affiliative physical contact and close proximity both before and after the competition, but not during a brief intervening cooperative task. These results suggest that in the face of competing self-interests, physical contact and close proximity facilitate repair of males’ more than females’ valuable relationships.

## Introduction

Competition refers to a contest between individuals over physical or social resources that enhance survival and reproductive success. In social species, competition can damage the relation between opponents. When that damage occurs to a valuable alliance, allies can no longer cooperate efficiently to attain mutual goals. Maintenance of cooperative alliances therefore necessitates learning to repair the damage that competition inflicts on relationships^1–4^.

Numerous studies document the mechanisms by which non-human social animals who form cooperative alliances repair their bonds following a competition. Affiliative physical contact and close proximity have been found to be the main links between competition and subsequent cooperation^1,2,4^. These behaviors allow two individuals who have mutually benefitted from prior cooperation to suppress their competitive tendencies once a contest has been decided, thereby permitting continued cooperation.

Studies of conflicts in non-human animals demonstrate that when affiliative behaviors occur following a contest, they typically appear within the first five minutes following the outcome^1,5^. Reconciliation between two former opponents therefore is defined as occurring when affiliative behaviors between two individuals increase within a short time post-conflict (PC), which is often compared to a matched control (MC) setting^1^.

Using the PC-MC method, affiliative behaviors following conflicts have been documented in all major taxa in the primate order including more than two dozen non-human primate species as well as in a number of other social mammals^2,4,6^. Species often differ in the percentage of conflicts that they reconcile. For example, even within the genus, *Macaca*, the proportion of contests that is reconciled ranges from a low of 4% to over 60%^7^. Likewise, within the genus, *Pan*, male chimpanzees (*Pan troglodytes*) reconcile a higher percentage of their conflicts than male bonobos (*Pan paniscus*)^5^. Further, sex differences frequently are found in rates of reconciliation within a species. Thus, male chimpanzees tend to reconcile more of their conflicts than female chimpanzees^8^. In contrast, female Hanuman langurs (*Semnopithecus (Presbytis) entellus entellus*) reconcile more conflicts than male langurs^9^.

Although species differ in the forms of affiliative behaviors that they utilize, physical contact and close proximity undergird most affiliative behaviors^4^. Some form of physical contact, such as grooming, embracing, linking arms, holding hands, kissing, patting, muzzling, cupping of bodily appendages, mounting, rubbing, nuzzling, and nudging, has been observed to indicate reconciliation across myriad species, for example chimpanzees^1^, bonobos^10^, gorillas (*Gorilla gorilla bereingi*)^11^, rhesus macaques^12^, stump-tailed macaques^13^, and bottlenose dolphins (*Tursiops truncates*)^14^ as well as in numerous other social non-primates^15^. Other affiliative behaviors that do not involve physical contact require close proximity, such as genital inspection in stump-tailed macaques^16^, lip-smacking in rhesus macaques^12^, subordinating the body in chimpanzees^1^, lying in close proximity in domesticated goats (*Capra hircus*)^17^, greeting and approaching in spotted hyenas (*Crocuta crocuta*), and synchronous swimming, following, and passing in bottlenose dolphins^14^.

Studies of the use of post-conflict affiliative behaviors using the PC-MC method in humans have examined only children. Human adults rarely exhibit conflicts in public and then generally unpredictably, rendering their natural conflicts difficult to examine systematically. Several studies suggest that similar to non-human animals, children in formal educational institutions exhibit affiliative behaviors following a conflict. Examples of children’s affiliative behaviors include hugging, holding hands, expressing empathy, apologizing, taking responsibility, smiling, offering objects, singing together, making a joke, assuming a fantasy role, and extending an invitation to play^18–20^. Across diverse cultures, no gender differences have been found in reconciliation of children’s conflicts. However, these conflicts occurred under supervision of adults who typically constrain the outcomes and aftermath.

Despite the ubiquity of human competition, there has been little scientific investigation of post-conflict behavior in human adults. It is well-established however that in humans by age three years^21,22^ through adulthood^23,24^, overt competition between same-gender peers occurs more frequently between human males than between females. This raises the important question of whether overt competition destroys human males’ valuable relationships, or if not, how males are able to repair damage inflicted on their bonds with same-gender peers^4^. The same critical question of whether, despite its relative rarity, overt competition between valuable female allies is repaired, and if so by what mechanisms.

Studies suggest that beginning in middle childhood and continuing into adulthood, competition inflicts greater damage on human female than male same-gender friendships. In middle childhood and adolescence, in response to hypothetical conflicts with same-gender friends, compared to boys, girls report they would be more likely to terminate the friendship^25,26^. At these same ages, compared to males, females also report that more of their actual same-gender friendships ended in response to conflicts with same-gender friends^27–29^. In experimentally created groups of four female classmates in middle childhood, competition for the leadership role elicited greater signs of behavioral discomfort in female than male groups^30^. In a role-playing scenario in which a young adult participant imagined a serious conflict with an actual same-gender friend, then confronted a same-sex experimenter who represented the friend, heart rates of young women during the confrontation rose more than those of young men. These women also reported that they would reconcile less rapidly than the men with their same-gender friend^31^. Moreover, following an actual conflict with a same-gender roommate, female college students at several institutions were more likely than their male counterparts to switch roommates^32^. Finally, subjective reports regarding competition with same-gender colleagues at work coupled with several experiments that induced competition between pairs also demonstrated that compared to men, women reported that competition damaged their same-gender relationships to a greater extent^33^.

One proximate explanation for the documented gender differences in the consequences of competition on same-gender relationships is that affiliative behaviors that can repair the bond are utilized more by human males than females in a competitive context. For example, following the outcome of a same-gender sports competition, across diverse cultures human males engaged in more extended affiliative physical contact than females^34^. Other studies likewise indicate that team sports competitions elicit more affiliative physical contact between male than female opponents^35^. Physical sports however have played a larger role in males’ than females’ lives for millenia^36^. Therefore, results may not apply to other forms of overt competition.

It is however plausible that human females may be less able than males to repair the damage inflicted on same-gender relationships by overt competitions that do not involve physical sports. This may stem from a lack of experience as females at all ages universally are less likely than males to compete^37^ and hence to obtain practice repairing the damage that competition inflicts on relationships. Consistent with this, a year-long study of children on playgrounds demonstrated that compared to girls, boys were better able to resolve their conflicts and resume their activities^38^. This prompted the conclusion that boys gain more experience with conflict resolution beginning in middle childhood, but it was based entirely on observations of boys engaged in physical sports.

One means to examine systematically gender differences in response to competition is to engage same-gender friends in a competition, then examine affiliative behaviors before and after the competition. Given that physical contact and close proximity, are considered the most critical affiliative behaviors, exhibition of these behaviors would suggest that individuals have the toolkit necessary to repair the damage to the friendship. Should males utilize affiliative behaviors more than females, this would suggest that males are better prepared to resolve conflicts.

A potential confound with this measure is the possibility that it might reflect general differences in the frequency with which affiliative behaviors are exhibited. However human females are commonly considered the more affiliative or communal sex, in contrast to males who are characterized as more independent and agentic^39,40^. More specifically, when sex differences in affiliative behaviors are found, cross-cultural studies utilizing naturalistic observations in homes and public spaces overwhelmingly demonstrate that human females are more likely than males to engage in affiliative physical contact with kin and non-kin and less likely to avoid affiliative physical contact^35,41,42^. By infancy and continuing through adulthood, human females spend more time than males in friendly physical contact overall, particularly with same-gender individuals^42^. In one study of 3 to 11 year old children from six diverse cultures, girls initiated and sought physical contact more than boys with all individuals^43^. Further, across diverse cultures adult females report being touched on more parts of their bodies than males and feeling more comfortable than males with physical contact from kin and friends of both genders. No sex differences in touching are reported with acquaintances or unfamiliar individuals of either gender.

To test the hypothesis that competition produces differing affiliative responses in males and females outside of physical sports, we created a standardized one-on-one contest testing different forms of real world knowledge with a randomly determined winner and loser. To ensure the motivation to repair any damage inflicted by the competition would be present, we recruited pairs of same-gender friends to compete. Finally, to maximize the chances of pairs exhibiting affiliative behaviors following the competition, as soon as the competition ended and the prize money was handed to the winner, we asked the two friends to sit side-by-side for 480 s. We coded on a second-by-second basis the first 300 s, the standard interval by which affiliative behaviors following competitions appear in a variety of species^1,2,5^.

We also asked all pairs first to relax in the identical position for 480 s preceding the competition, and for the purposes of comparison again coded the first 300 s. The pre-competition relaxation period served to examine an intriguing possibility- that anticipatory affiliative behaviors may be exhibited prior to a known incipient contest. In non-human primates in which individuals expect an impending competition, several studies show that affiliative behaviors are exhibited prior to the contest. Anticipatory affiliative behaviors have been observed in bonobos^44^ and chimpanzees^45,46^ who engage in increased rates of genital rubbing, play or grooming when they are aware that food will be delivered shortly. Anticipatory affiliation has not however been studied systematically in humans.

Immediately following the competition, each pair was asked to engage in a 150 s cooperative task in which the pair needed to closely coordinate their actions to obtain points. The cooperative task served to reinforce the bonds between the friends in the pair just prior to the competition. We also used it as a comparison to the more relaxed periods preceding and following the competition.

The competition was designed to be non-gender typed and meaningful to the participants so that the outcome would be important. Verbally delivered questions measured skills that are critical to success in modern life, including financial, social, emotional, and practical as well as mathematical and verbal forms of intelligence. The relaxation periods immediately following and preceding the competition were videotaped by a camera positioned four feet behind the participants’ backs to reduce self-consciousness. During the relaxation periods, each individual was provided with identical materials so that they could choose to engage in reading, coloring, or playing cards by themselves or as a pair.

Joint physical contact and close proximity were coded separately for the first 300 s of the relaxation periods following and preceding the competition. During the cooperative task, physical contact rarely occurred, so only close proximity was coded. Time spent in joint physical contact was defined as the total amount of time physical contact occurred between any parts of the two participants’ bodies. Time spent in close proximity was defined as the total amount of time each focal participant spent across an imaginary two-dimensional plane bisecting the two participants’ chairs without making physical contact. No two chairs were positioned more than 12 cm. apart, so that when one participant crossed the plane, the two participants were in close proximity.

## Results

For each pair, we tabulated both the total time the pair was in joint physical contact and the total time each participant was in close proximity to the other participant. We calculated mean total times of affiliative behaviors as a function of period (pre-competition relaxation period, post-competition relaxation period, and cooperation task) and gender (see Table 1). During the pre- and post-competition phases, the values of joint physical contact ranged from 0 to 271 s. A number of pairs did not engage in any physical contact and others engaged in extensive physical contact and hence the data for joint physical contact were not normally distributed. Therefore, after adding a one to each pair’s total time of physical contact, we applied a natural logarithmic transformation to construct the final measures of pre-competition and post-competition joint physical contact. The values of time spent in close proximity in contrast were normally distributed and ranged from 0 to 298 s for each participant. For the cooperative task, the values of time of close proximity also were normally distributed and ranged from 0 to 150 s for each participant.

**Table 1:** Mean time (SE) of affiliative behaviors by gender and period (Pre-competition, Post-competition, Cooperation).

	Pre-competition		Post-competition		Cooperation*
	Close proximity	Physical contact	Close proximity	Physical contact	Close proximity
Females (*n* = 21)	60.5 (10.7)	5.2 (2.0)	70.8 (10.9)	2.6 (1.0)	107.9 (9.8)^a^
Males (*n* = 20)	115.3 (11.6)	32.0 (14.2)	129.8 (13.4)	16.4 (6.4)	118.0 (9.4)

^a^This result is based on *n* = 20 pairs due to a video error.

*Duration of cooperation was 150 s while duration of pre- and post-competition was 300 s.

To examine gender differences in time spent in joint physical contact in preparation for and in the aftermath of a competition, a repeated measures analysis of variance (ANOVA) was conducted with time period (pre-competition versus post-competition) as the repeated factors and gender of pair as the independent variable. Consistent with the hypothesis, results yielded a significant effect of gender on time spent in joint physical contact, *F*(1, 39) = 8.99, *p* = 0.005, with male friends spending more time than female friends in joint physical contact. Additionally, using chi-square analyses, we calculated the percentage of pairs who engaged in no physical contact in the pre-competition period (33.3% or 7/21 of female pairs versus 10.0% or 2/20 of male pairs, *X*^2^(1) = 3.42, *p* = 0.065) and in the post-competition period (33.3% or 7/21 of female pairs versus 5.0% or 1/20 of male pairs, *X*^2^(1) = 5.80, *p* = 0.016). Only in the post-competition phase did significantly more female than male friends avoid physical contact completely, although the same trend existed in the pre-competition phase.

To compare gender differences in time spent in close proximity, a repeated measures analysis of variance (ANOVA) was performed with period (pre-competition versus post-competition) and status (winner versus loser) as the repeated factors and gender of pair as the independent variable. Again, in accord with the hypothesis, results yielded a significant effect of gender on time spent in close proximity, *F*(1, 39) = 16.94, *p* < 0.001, with male friends spending more time than female friends in close proximity (see Table 1). When both the analysis of time spent in joint physical contact and in close proximity were conducted with friendship quality as a co-variate, the results were unchanged.

To investigate whether time spent in affiliative behaviors before the competition predicted time spent in affiliative behaviors following the competition, we performed regression analyses separately on time spent in joint physical contact and time spent in close proximity in the post-competition phase as dependent variables and gender and pre-competition levels fully crossed as independent variables. For the regression on time spent in joint physical contact, there were significant effects of pre-competition level, *b* = 0.33, *t*(37) = 3.03, *p* = 0.005, gender, *b* = −0.34, *t*(37) = −2.09, *p* = 0.043, and a significant gender X pre-competition level interaction, *b* = −0.29, *t*(37) = −2.71, *p* = 0.01. The relation between pre- and post-competition levels was significantly higher for male friends, *r*(18) = 0.79, *p* < 0.001 than for female friends, *r*(19) = 0.046, n.s. Thus, pre-competition levels of time spent in joint physical contact predicted post-competition levels of time spent in joint physical contact for male friends only. For the regression on time spent in close proximity, there was a significant effect of pre-competition level, *b* = 0.45, *t*(37) = 2.73, *p* = 001, and a marginal effect of gender, *b* = −34.4, *t(*37) = −1.87, *p* = 0.070. In this case, time spent in close proximity pre-competition predicted time spent in close proximity post-competition. The relation between pre- and post-competition levels were only marginally stronger between male friends compared with female friends. Time spent in close proximity preceding the competition significantly predicted time spent in close proximity following the competition for male pairs, *r* (18) = 0.46, *p* < 0.05, and marginally significantly predicted close proximity following the competition for female pairs, *r* (19) = 0.39, *p* < 0.10.

To examine whether the observed gender differences occurred in a different context, we analyzed time spent in close proximity (joint physical contact virtually never occurred) during the cooperative task. An independent samples *t*-test was conducted on time spent in close proximity with gender of pair as the independent variable. No gender differences were obtained, *t*(39) = 0.78, *p* = 0.44, two-tailed. Given the different nature of the contexts, the absolute levels cannot be compared.

## Discussion

This study shows that male friends engaged in more physical contact and remained in closer proximity than females both before and after a non-physical competition. These results extend prior findings that were limited to post-competition affiliative behaviors in the context of individual and team sports competitions^34,35^. Further, for male pairs, time spent in joint physical contact prior to the competition strongly predicted time spent in joint physical contact following the competition.

This result is consistent with the hypothesis that male pairs are better able than female pairs to engage in affiliative behaviors that can repair the damage that a contest inflicts on a relationship. By exhibiting friendly behaviors both before and after a contest, male pairs may efficiently communicate to one another their willingness both to accept the outcomes of the competition and to resume the cooperative facet of their relationship. Positioning the body in close proximity to and physically making contact with a future or former opponent inherently contains risk should an opponent wish to take preemptory or retaliatory action surrounding a contest. Engaging in these two affiliative behaviors demonstrates a powerful degree of trust in a prospective or former opponent^47^.

Given the consistent findings that girls and women engage in affiliative physical contact more than boys and men^39–42^, the results cannot be explained by males’ greater usage of physical contact or close proximity. Rather the results are consistent with findings that competitions are the only contexts that consistently produce higher rates of physical contact between male than female same-gender peers^23,41^.

From an adaptive perspective, in humans, males may be more likely to repair alliances rapidly than females in order to prevail in inter-group conflicts which require intra-group cooperation^48,49^. A damaged valuable relationship would hinder intra-group cooperative efforts, increasing the vulnerability of the community. Evidence from a number of studies that beginning in middle childhood, human males repair valuable relationships following conflicts faster than females do supports this interpretation^14,20–22^.

These results are particularly interesting as no gender differences are observed in young children in use of affiliative contact after competition^17^ Given the well-documented fact that males engage in more competition than females at all ages^22,23^, this suggests that adult males’ increased use of affiliative contact is at least partly the result of experience with the consequences of the potentially negative outcomes of competition, as others have proposed^38^.

One of the more interesting aspects of these results is the increased affiliative behavior shown by males prior to competition. This suggests that males who are anticipating a competition signal to their partners that they do not intend to inflict damage on their relationship. To our knowledge, this is the first time that pre-competition behavior has been investigated in humans. It is difficult to examine this behavior because many contests may not be predictable. Anticipatory affiliative behaviors can occur only in contexts in which individuals are aware that they will shortly become competitors, such as chimpanzees approaching a fruit tree or recently killed prey, or young children approaching a new toy or favored space or person^34,42^. Future studies of predictable contests, such as when individuals know that food, mates, or other valuable physical or social resources will materialize shortly, or for humans during scheduled competitions, will permit systematic examination of the role of pre-competition affiliative behaviors.

The cooperative task also was not an ideal comparison condition, as the participants remained aware that they imminently would have to compete. Furthermore, the relaxation periods were much less structured than the cooperative task, so that the cooperative task provided differing affordances which clearly influenced the behaviors of participants. Nonetheless, the cooperative task demonstrates that the male pairs did not consistently engage in more close proximity than the female pairs.

Limitations of the research include the fact that participants were required to sit on the same side of a table preceding and following the competition which constrained their behavior. However, participants were told they were free to adjust the positioning of their chairs which they did. Additionally, the competition itself and the random choice of the winner and loser may have generated artificial responses or impacted each gender differentially.

Additional affiliative behaviors that do not require physical contact or close proximity, such as spoken or written words or complex non-verbal gestures uniquely exhibited by humans, also merit investigation as tools to repair relationships following competition. It is also possible that physical contact by males might serve an agentic as well as a purely friendly function. However, physical contact, and by extension close proximity, is believed to take communicative primacy over other forms of both non-verbal and verbal interactions^47^, partially because it exposes participants to the possibility of potentially dangerous physical retaliation.

We do know that virtually every pair of friends spent the relaxation period following the competition dissecting the reasons for the outcome, indicating that the competition strongly influenced all the participants. The camera also may have affected participants’ behaviors. Review of the videotapes however shows that as soon as the experimenter left the room, participants relaxed and seemed oblivious to being recorded as indicated by a number of pairs exchanging sensitive information that most adults would not share publicly. By not looking towards the camera, participants were able to remain anonymous. Replication of the current results however will require additional investigations that includes other types of conflicts and additional contexts, with peers who differ in gender and sexual identity, as well as with kin and heterosexual and homosexual romantic partners.

An intriguing interpretation of the current findings is that the observed gender difference in physical affiliation could arise not only through male friends increasing, but also through  female friends decreasing their affiliative behavior surrounding the competition, possibly due to the differential impact of competition. Research on reconciliation in non-human animals^1,2^ using the PC-MC method demonstrates that affiliative behavior increases more following a competition relative to a matched setting, consistent with the interpretation that male friends increased their affiliative behavior surrounding the competition. Further, the high levels of physical contact and close proximity that we observed for male friends were striking. Nonetheless, it is possible that female friends reduced their level of affiliative behavior or some combination of males’ increasing and females’ decreasing their affiliative behaviors occurred. Findings that one-third of female pairs never made any physical contact would support an interpretation that females may be avoiding one another.

Replication of these results may help explain why unrelated human males compete more readily than unrelated females. Compared to women, men may be better able to employ a toolkit of affiliative behaviors, similar to those utilized by many other species, to both engage in and disengage from contests with valuable partners. This would facilitate the ability of unrelated men to further their own self-interests in competitive contexts without losing allies. It would also permit men to coordinate rapidly in inter-group conflicts.

## Methods

### Participants

This study was approved by the Institutional Review Board of Emmanuel College. The methods were carried out in accordance with the relevant guidelines and regulations. Informed consent was obtained from all participants. Participants consisted of 41 pairs (82 individuals) of same-gender, heterosexual friends of whom 75% were white with the remaining individuals Black, Asian, and Hispanic. The ages in years were *M* = 19.07 (*SD* = 0.93, *n* = 21 pairs) for females and *M* = 19.98 (*SD* = 1.66, *n* = 20 pairs) for males. For the cooperation task, one pair of females was lost due to failure of the video equipment. Prior to arrival, participants were screened to ensure they were same-gender, heterosexual friends. To assess the quality of their friendship, each participant rated their relationship with their partner on a 100-point scale with 1 = enemy, 50 = average familiar person, to 100 = very best friend. Averaging both friends’ evaluations produced an average friendship rating of 84.76 (*SD* = 11.01) for the 21 female pairs and 85.85 (*SD* = 7.02) for the 19 male pairs (one pair’s ratings were lost), *t* (39) = 0.37, n.s., indicating that individuals had formed meaningful friendships.

### Procedure

Pairs of same-gender friends were recruited to participate in a study on cooperation and competition. All pairs participated in a 480 s pre-competition relaxation period followed by a 150 s computer-based collaboration exercise, a saliva collection, a 1200 s (20-minute) competition conducted by an experimenter, a second saliva collection, then a 480 s post-competition relaxation period. Video was recorded during the relaxation periods and the cooperative task. The experimenter was absent from the room during the relaxation periods and was out of sight during the cooperative task. The saliva was collected as part of a larger study that is ongoing.

**Figure 1:**
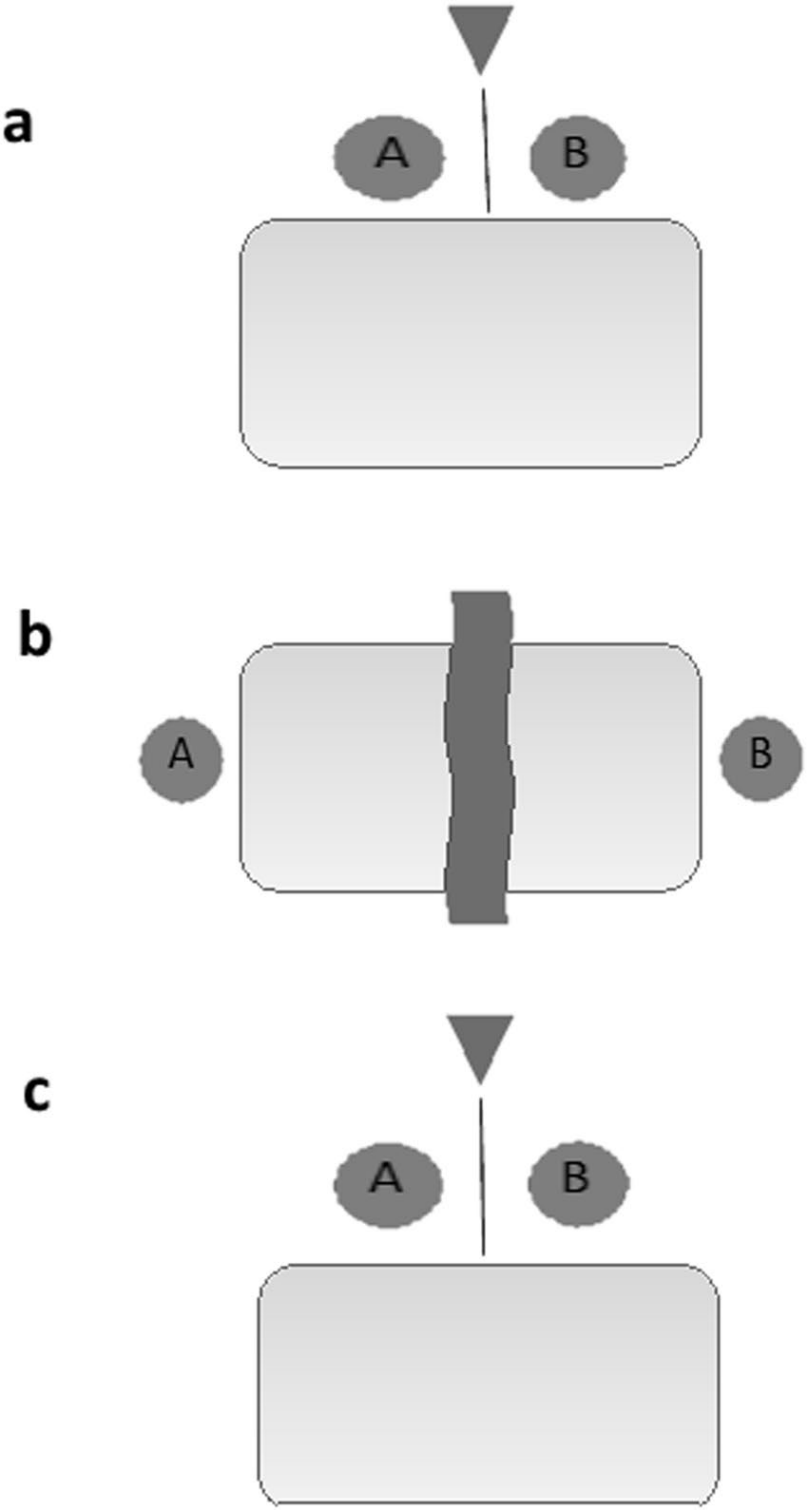
Disposition of pairs. Disposition of pairs during the pre-competition (**a**), the competition (**b**) and the post-competition (**c**) phases. Imaginary center line used to code close proximity is indicated in (**a**) and (**c**).

Once the participants arrived at the laboratory, the study was re-explained, consent forms were signed, and the participants were reminded that they would be competing against one another and cooperating and that a camera situated behind them would record their behavior. For the pre-competition and post-competition relaxation periods, the participants were asked to position their chairs on the same side of a 1.2 m.-long rectangular table (see Fig. 1). All chairs were positioned so that the inside arm rests were within 12 cm of each other. The experimenter then placed in front of each participant a picture book of photographs from around the world, a deck of playing cards, and blank coloring paper with colored markers. Prior to each relaxation period, each pair was informed that they could engage in any or no activity, that they could communicate if they so desired, and that the experimenter would return in 8 minutes. They were asked to remain seated in their chairs unless they needed to stand up to stretch or realign their chairs. A camcorder on a 1.2 m.-tripod was positioned four feet behind the backs of participants, so as to reduce the self-consciousness of the participants. To remain anonymous, participants were told they should not turn around to face the camera.

After the first relaxation period, participants collaborated on a computer-generated cooperative task for 150 s. For the cooperative task, the experimenter placed a laptop computer on the table in between the two participants. Participants were informed that those pairs of friends who scored in the top 5% on the cooperative task would receive $100 per pair at the conclusion of the study. The cooperative task consisted of 25 lengthy and complex words, each one presented one at a time, at the top of the screen. For each word, one participant was required to count the number of letters in a word that were between A and M, while the other participant needed to select which of two sets of four letters matched the first four letters of a word in reverse order. In order to make a correct response, the two participants were required to enter their responses simultaneously. Thus, continuous communication was necessary to ensure coordination of responses. After the cooperative task, participants provided a saliva sample as part of an ongoing study.

Next, the experimenter began the 1200 s (20-minute) competition. Participants were asked to position their chairs at opposite sides of the rectangular table, and a curtain was extended between them so that they could not see one another (Fig. 1). To ensure that participants were invested in beating their opponent, each participant was asked to circle their own four areas of greatest knowledge, and their partners’ three weakest areas from the following categories that were explained as critical to success in life: personal finance, geography, memory of a newspaper item, anagrams, health, probability, social acuity, biology, and government. The experimenter then indicated that throughout the competition the questions would become biased in favor of the player who was winning by choosing questions that favored them and hurt their opponent. The competition consisted of 30 questions selected from all nine categories. For each question, participants had 20 seconds to record their answer on paper and hand it to the experimenter. They were told that they should give their “best intuitive answer”, and that the participant whose answer was closest to the correct one would win one point. Participants were required to write an answer to each question.

After every 5 problems, the experimenter announced the score of each participant and reported which participant was winning. Undisclosed to the participants, at the start of the competition the experimenter randomly chose a winner and loser, and throughout the competition ensured that the final score reflected a clear winner and loser. Therefore, regardless of the participants’ answers to the questions, the experimenter reported pre-determined scores throughout the competition which began to diverge in favor of the winner in a standardized fashion [2 (winner): 3 (loser), 7 (winner): 3 (loser), 10 (winner): 5 (loser), 14 (winner): 6 (loser), 17 (winner): 8 (loser), 20 (winner): 10 (loser)]. Before beginning the competition, the participants were informed that the payoff would depend on the number of points won, with the possibility of a bonus payout of $5 if one participant bested the other participation by a ratio of 2:1. At the end of the competition, the experimenter warmly congratulated the winner and handed him/her $25, then quickly handed $5 to the loser. The winner then was asked to sign a form, in the name of both participants, to confirm payment.

Immediately following the awarding of the money, participants provided a second saliva sample. They then were asked to move their chairs back to their original positions during the first relaxation period in which they sat side-by-side (Fig. 1). During the post-competition relaxation period, the experimenter provided the participants with the identical materials and instructions as for the pre-competition relaxation period, then left the room. At the end of the experiment, a de-briefing procedure was carried out in which the participants were informed of the random assignment of winners and losers and were asked to split the money equally while the purpose of the study was explained in greater detail.

### Coding

For the competitive task, the two measures, joint physical contact and spatial proximity, were coded during the first 300 s of the pre-competition and post-competition relaxation periods. Joint physical contact was coded when the two participants touched one another’s bodies regardless of which bodily parts made contact. No instances of non-affiliative contact occurred, so physical contact was not sub-divided into differing forms. Close proximity was coded separately for each participant. Close proximity was defined as occurring when the focal participant reached across the imaginary plane bisecting the two participants’ chairs into the spatial field of the second participant (see Fig. 1) but no physical contact occurred. Close proximity was coded separately for the winner and loser of the competition. For the cooperative task, close proximity was coded for the entire 150 s separately and averaged for each pair.

The software program BORIS (Behavioral Observation Research Interactive Software) [http://www.boris.unito.it/] recorded start and stop times for each behavior with total frequency of each behavior summed to create the final scores for each period. For the pre-competition and post-competition periods, coding began after the experimenter had left the room and continued for 300 s. Joint physical contact was coded in one pass. Close proximity was coded first for the participant seated on the left, then independently for the participant seated on the right. Close proximity was similarly coded for the entire cooperative task. Two coders rated all videotapes. To assess reliability, correlation coefficients between each coder’s ratings were computed. For the pre- and post-competition periods, the reliability was as follows: joint physical contact pre-competition, *r* = 0.91, joint physical contact post-competition, *r* = 0.84, close proximity pre-competition left position, *r* = 0.93, close proximity pre-competition right position, *r* = 0.90, close proximity post-competition left position, *r* = 0.83, and close proximity post-competition right position, *r* = 0.91. To enhance the validity of all measures, both coders’ ratings were averaged for each measure, yielding spearman-brown effective reliabilities of 0.95, 0.91, 0.96, 0.95, 0.91, and 0.95, respectively. Close proximity was re-classified into close proximity for the winner and loser for the post-competition period.

For the cooperative task, the reliability was calculated for close proximity in the same manner as for physical contact yielding the following correlation coefficients: close proximity left position, *r* = 0.86, close proximity right position, *r* = 0.71. To enhance the validity of both measures, the two coders’ ratings were averaged yielding spearman-brown effective reliabilities of 0.92 and 0.83, respectively.

### Data Availability

The datasets generated during and/or analysed during the current study are available from the corresponding author on reasonable request.
